# Test and Analysis of High-Permeability Material’s Microstructure in Magnetic Shielding Device

**DOI:** 10.3390/ma16113956

**Published:** 2023-05-25

**Authors:** Weiyong Zhou, Jinji Sun, Bangcheng Han, Jianyi Ren, Yifei Li

**Affiliations:** 1 School of Instrumentation and Optoelectronic Engineering, Beihang University, Beijing 100191, China; weiyong_zhou@buaa.edu.cn (W.Z.); sunjinji@buaa.edu.cn (J.S.); 2 Hangzhou Innovation Institute, Beihang University, Hangzhou 310051, China; 3 Zhejiang Provincial Key Laboratory of Ultra-Weak Magnetic-Field Space and Applied Technology, Hangzhou 310051, China; 4 Hefei National Laboratory, Hefei 230088, China; 5 Ningbo Institute of Materials Technology and Engineering, Chinese Academy of Sciences, Ningbo 315201, China; liyifei@nimte.ac.cn

**Keywords:** high-permeability material, microstructure, magnetic shield device, magnetic property

## Abstract

The magnetic shielding device is used to provide an extreme weak magnetic field, which plays a key role in variety of fields. Since the high-permeability material constituting the magnetic shielding device determines the magnetic shielding performance, it is important to evaluate the property of the high-permeability material. In this paper, the relationship between the microstructure and the magnetic properties of the high-permeability material is analyzed using minimum free energy principle based on magnetic domain theory, and the test method of the material’s microstructure including the material composition, the texture and the grain structure to reflect the magnetic properties is put forward. The test result shows that the grain structure is closely related to the initial permeability and the coercivity, which is highly consistent with the theory. As a result, it provides a more efficient way to evaluate the property of the high-permeability material. The test method proposed in the paper has important significance in the high efficiency sampling inspection of the high-permeability material.

## 1. Introduction

The magnetic shielding device is used to reduce the ambient magnetic interfering signals (including the geomagnetic field and the ambient magnetic field fluctuations) significantly, by which, an extreme weak, high uniform, small time drifts, and low noisy magnetic field could be obtained [1,2,3]. The demands for the magnetic shielding device come from a variety of fields such as fundamental physics [4,5], biomedical science [6,7], underground survey [8], space research [9], and industrial applications [10]. One typical application of a magnetic shielding device in industry is the high sensitivity magnetic detection based on the effect of spin change relaxation free (SERF). With the advantages of non-radiation, non-contact, high sensitivity, small volume, and low cost [11,12], the high sensitivity magnetic detection brings broad application prospect and high commercial value, especially in the areas of magnetocardiogram (MCG) and magnetoencephalography (MEG) [13]. The magnetic shielding device plays a key role to ensure the accuracy and sensitivity of the magnetic detection.

Magnetic shielding device is usually constructed by the high-permeability materials based on the effect of flux shunting [14], and high-conductivity materials based on the effect of eddy current [15]. Among them, the property of the high-permeability materials (a kind of soft magnetic material) always determines the performance of the magnetic shielding device in most applications [16,17]. In order to achieve a better magnetic shielding performance, it is important to obtain the complete property of the high-permeability material before the construction of the magnetic shielding device.

The most concerned magnetic properties of the high-permeability material related to magnetic shielding performance are the initial permeability, the coercivity, the maximal permeability, the saturation magnetic induction, and the remanence [18], which could be obtained by a standard soft magnetic testing instrument such as MATS-3000S. The measuring principle by a standard soft magnetic testing instrument is based on the electromagnetic induction, and the magnetic properties could be obtained by the hysteresis loop [19]. The test ring needs to be wound evenly by coils to generate the excitation magnetic field and obtain induced voltage for the calculation of magnetic induction intensity [20], which brings the testing process inconvenience and low efficiency. In some recent studies, the probe and H-coil without winding were used to obtain the magnetic induction intensity and magnetic field intensity, respectively [17,21]. However, the structure used to form the closed magnetic loop is still complex and the accuracy in low magnetic field intensity is hard to maintain.

According to the research on the soft magnetic materials, the magnetic properties could be explained by the magnetic domain theory [22,23,24,25]. The minimum free energy principle (MFE) [26] based on magnetic domain theory is usually used to calculate the magnetic properties of soft magnetic materials, such as the initial permeability and the coercivity. The initial permeability and the coercivity could be reflected by the anisotropy and the magnetostriction (related to the material composition and texture), the grain structure, the defect, the stress, etc. [18,27]. The MFE method is already used to calculate the influence of stress and defects, but rarely used to analyze the pinning effect of the grain boundary. Since the measurement of the microstructure is more direct and efficient, we are motivated to explain the mechanism of microstructure in theory and explore a new test method for the high-permeability material focusing on the microstructure.

In this paper, a test method of high-permeability material’s microstructure including the material composition, the texture, and the grain structure is proposed, and the relationship between the microstructure and the magnetic properties of the high-permeability material is analyzed and calculated through the magnetic domain theory, which is verified by the experiment. The main contributions are listed as follows: (1) a novel test method of the high-permeability material’s microstructure to reflect the high-permeability material’s magnetic properties is proposed for the first time. (2) MFE based on magnetic domain theory to explain the pinning effect of the grain boundary is firstly introduced to analyze the relationship between the microstructure and the magnetic properties. (3) An effective formulation of etchant for permalloy corrosion is obtained. (4) Compared with typical magnetic testing methods, the proposed method of microstructure to obtain the magnetic properties will promote the detection efficiency and reduce the detection complexity.

This paper is organized as follows. The theory reflecting the relationship between the microstructure and the magnetic properties of the high-permeability material is described in Section 2. The test of magnetic properties and microstructure of the high-permeability material is recorded in Section 3. The discussion of the test results when compared with the theory is arranged in Section 4. Through the experiment results, the relationship between the microstructure and the magnetic properties is proved to be clear and credible, which provides a new approach to evaluate the property of the high permeability material.

## 2. Theory of Microstructure’s Magnetic Properties

The magnetic domain theory is widely applied to explain the magnetic properties’ mechanism of the high-permeability material [24,28,29], which follows the minimum free energy principle. For a ferromagnet with unit volume, the total free energy $F_{T}$ could be described as follows:

(1) $$F_{T}=F_{H}+F_{\sigma }+F_{w}$$

where $F_{H}$ is the external magnetic field energy, $F_{\sigma }$ is the magnetoelastic energy and $F_{w}$ is the domain wall energy. The most commonly used high-permeability material in the magnetic shielding device is the large-grained material such as permalloy, and the grain size *D* follows the relation $D\gg L_{ex}$, where $L_{ex}=\sqrt{A/K_{1}}$ is the ferromagnetic exchange length, *A* is the exchange energy constant and $K_{1}$ is the anisotropy constant. The magnetic properties of large-grained material could be explained by the domain wall extension theory. During the process of domain wall expansion, the change of domain distribution follows the minimum free energy principle:

(2) $$\delta F_{T}=\delta F_{H}+\delta F_{\sigma }+\delta F_{w}=0$$

Ignoring the change of magnetoelastic energy near the domain wall, the magnetization equation could be written as:

(3) $$-\delta F_{H}=\delta F_{w}$$

For the typical 180° domain wall, the variation of the external magnetic field driving energy $\delta F_{H}$ and the domain wall energy $\delta F_{w}$ could be expressed as:

(4) $$\delta F_{H}=-\mu_{0}M_{s}Hcos0^{\circ }-\left(-\mu_{0}M_{s}Hcos180^{\circ }\right)=-2\mu_{0}M_{s}H$$

(5) $$\delta F_{w}=\frac{\partial r_{w}}{\partial x}$$

where $\mu_{0}$ represents the permeability of vacuum, $M_{S}$ is the saturated magnetization and *H* is the external magnetic field intensity, $r_{w}$ is the domain wall energy and *x* is the displacement of domain wall expansion in one dimension. Then, the displacement equilibrium equation for the 180° domain wall could be obtained from Equation (2) as follows:

(6) $$2\mu_{0}M_{s}H=\frac{\partial r_{w}}{\partial x}$$

The differential of the 180° domain wall energy $\frac{\partial r_{w}}{\partial x}$ could be expressed as [29]:

(7) $$\frac{\partial r_{w}}{\partial x}=A{\left(\frac{d\phi }{dx}\right)}^{2}+K_{1}\sin^{2}\phi ,x\in \left(-\infty ,+\infty \right)$$

where ϕ is the rotation angle of the domain and

(8) $$\phi =2arctan(e^{\frac{x}{L_{ex}}})$$

The typical parameters to evaluate the magnetic properties of the magnetic shielding device include the coercivity and the initial permeability. The coercivity is produced when the variation of the domain wall energy exceeds the peak value, which makes the magnetization process irreversible. The coercivity in the domain wall extension model $H_{c0}$ from Equation (6) could be expressed as follows:

(9) $$H_{c0}=\frac{1}{2\mu_{0}M_{s}}{\left(\frac{\partial r_{w}}{\partial x}\right)}_{max}=\frac{K_{1}}{\mu_{0}M_{s}}$$

For material with large grains, the magnetization process is determined by domain wall pinning at the grain boundaries, so the coercivity for material with large grains $H_{c}$ is obtained as follows:

(10) $$H_{c}=P_{H}\frac{L_{ex}}{D}H_{c0}=P_{H}\frac{\sqrt{AK_{1}}}{\mu_{0}M_{s}D}$$

where *D* is the grain size and $P_{H}$ is the correction factor for the domain size and the rotation angle of the adjacent magnetic domains.

The initial permeability is related to the magnetic susceptibility of the material. When the external magnetic field intensity varies δH, domain wall moves a distance of δx. According to Equation (6), the relationship of ΔH and Δx in the 180° domain wall could be written as:

(11) $$2\mu_{0}M_{s}\Delta H=\frac{\partial^{2}r_{w}}{\partial x^{2}}{|}_{x=x_{0}}\Delta x$$

where $x_{0}$ is the initial position of the domain wall, and the magnetization varies along with the domain wall displacement as:

(12) $$\Delta M=2M_{s}S\Delta x$$

where ΔH represents the variation of the magnetization and *S* is the area of the domain wall. Then, the initial susceptibility $\chi_{i0}$ could be obtained as:

(13) $$\chi_{i0}=\frac{\Delta M}{\Delta H}=4\mu_{0}M_{s}^{2}S/\left(\frac{\partial^{2}r_{w}}{\partial x^{2}}{|}_{x=x_{0}}\right)$$

It is not easy to determine the initial position in a domain, so the average variation rate is used as the approximate substitution as follows:

(14) $$\frac{\partial^{2}r_{w}}{\partial x^{2}}{|}_{x=x_{0}}\approx \left({\left(\frac{\partial r_{w}}{\partial x}\right)}_{max}-0\right)/\left(l_{d}/2\right)=\frac{4K_{1}}{l_{d}}$$

where $l_{d}$ is the width of the domain. In the unit volume, the area of domain wall could be expressed as $S=1/l_{d}$. According to Equation (13), the initial permeability of the domain $\mu_{i0}$ could be written as:

(15) $$\mu_{i0}\approx \chi_{i0}\approx \mu_{0}M_{s}^{2}/K_{1}$$

Considering the material with large grains pinning at the grain boundaries, the initial permeability $\mu_{i}$ could be obtained as follows:

(16) $$\mu_{i}=P_{\mu }\frac{D}{L_{ex}}\mu_{i0}=P_{\mu }\frac{\mu_{0}M_{s}^{2}D}{\sqrt{AK_{1}}}$$

where $P_{\mu }$ is the correction factor for the domain size and the rotation angle of the adjacent magnetic domains.

The relationship between the magnetic properties and the microstructure is established according to Equations (10) and (16), where the parameters *A*, $K_{1}$, and $M_{s}$ are related to the material composition and the texture, and *D* is related to the grain structure.

## 3. Test and Results

There is a clear correlation between the magnetic properties and the microstructure of large grains’ material in theory. This section introduces the testing method of the microstructure and explores the relation between the magnetic properties and the microstructure by experiments. The permalloy used in the magnetic shielding device is chosen as the material of the test samples with different annealing processes by different manufacturers. The information of the test samples are arranged in Table 1.

### 3.1. Magnetic Properties Test

**Figure 1:**
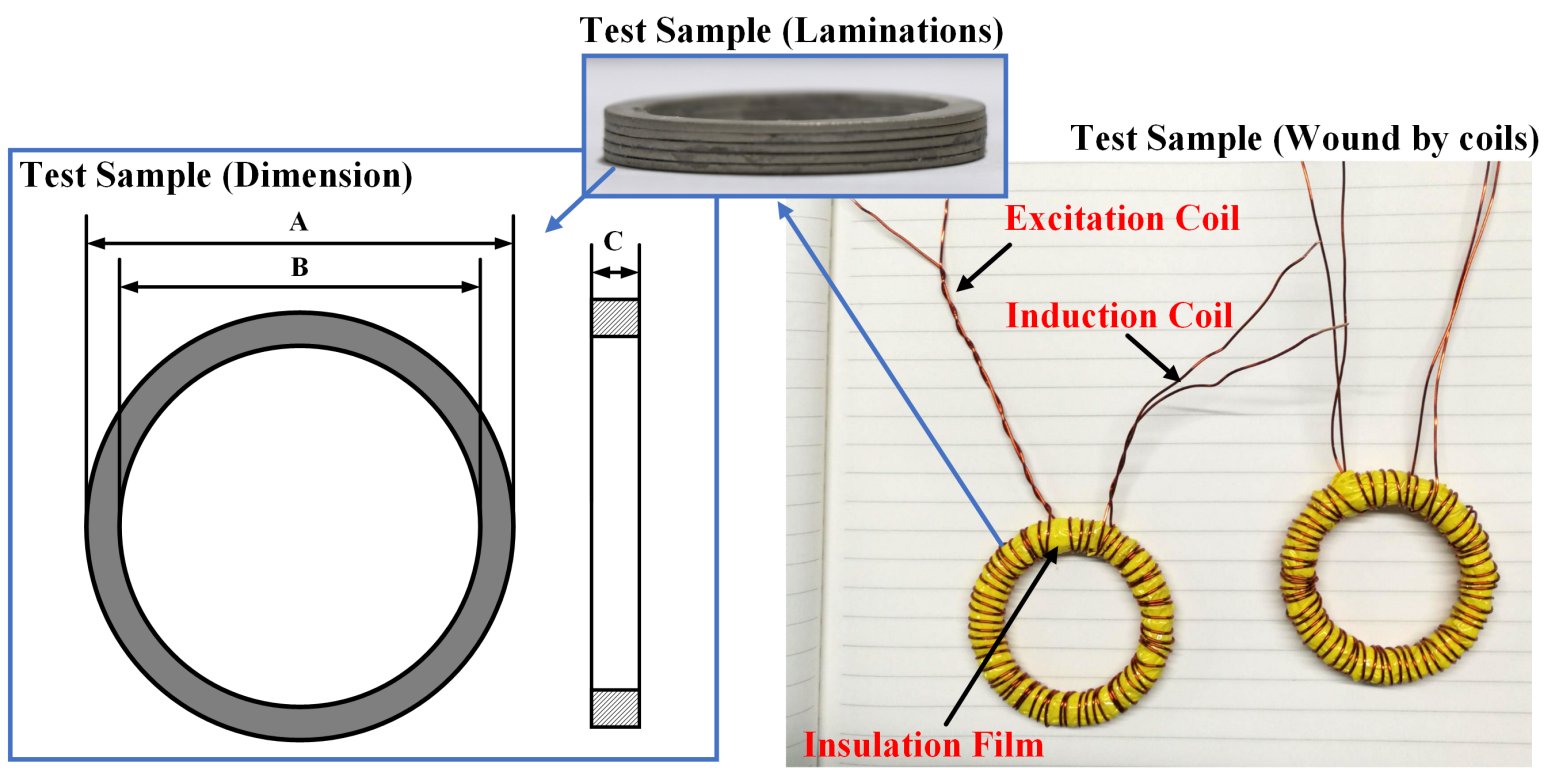
The schematic diagram of the test sample.

In order to obtain the magnetic properties, the test samples are processed into a circular shape to match the standard soft magnetic testing device (MATS-3000S). The measuring principle by the standard soft magnetic testing instrument is based on the electromagnetic induction, and the magnetic properties could be obtained by the hysteresis loop from the device. The test samples were wound evenly by the excitation coil to generate the excitation magnetic field and the induction coil to obtain induced voltage for the calculation of magnetic induction intensity, which is shown in Figure 1. The test sample, the excitation coil, and the induction coil are separated by the insulation film to avoid short circuiting. During the test, laminations are used to increase the signal strength of the induction coil. The test parameters for each sample is arranged in Table 2.

**Figure 2:**
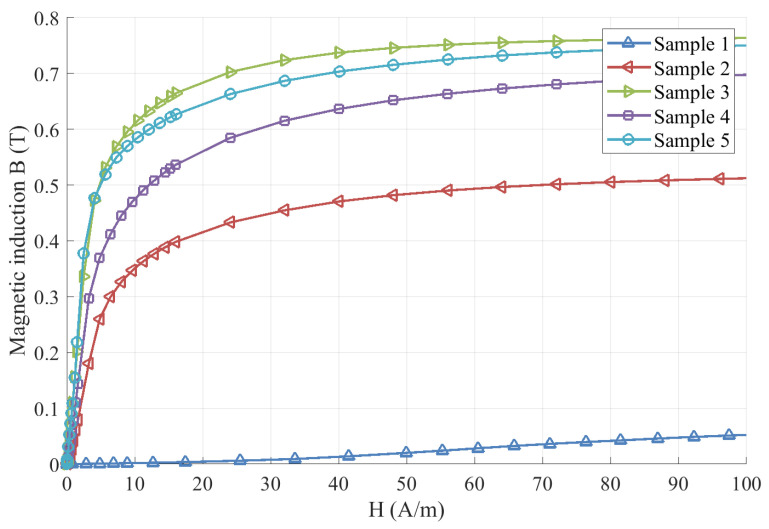
B-H curve of the test samples.

The test results including the initial permeability ($\mu_{i}$), the maximum permeability ($\mu_{m}$), the coercivity ($H_{c}$), the remanence ($B_{r}$), and the saturation magnetic induction ($B_{s}$) for each test sample are listed in Table 3 and the magnetization processes (B-H curve) for test samples in one measurement is are shown in Figure 2.

### 3.2. Microstructure Test

To verify the relation between the magnetic properties and the microstructure, the measurement of the material composition, the texture, and the grain structure is described in this section. The material compositions of samples were analyzed using a scanning electron microscope with energy dispersive spectroscopy (NOVA NANO SEM 200). The textures of samples were measured by an X-ray diffractomer (XRD-7000, Shimadzu, Kyoto, Japan) using CuKα radiation with a voltage of 40 kV and a current of 30 mA. The step size is 0.02°, the scanning speed is 2°/min, and the scanning range is 20∼90°. The grain structure is observed by a confocal laser scanning microscope(LEXT OLS5100, Olympus, Tokyo, Japan).

#### 3.2.1. Material Composition Test

The compositions of the test samples in Table 1 were tested using the scanning electron microscope (SEM). The testing procedures of material composition are listed as follows:

- Cut the test samples into appropriately sized pieces, then wash and dry the pieces for later use;
- Polish the surface of the sample pieces until they are smooth as a mirror;
- Place the test pieces in the SEM and observe them at magnification of 200 and 1600, respectively;
- Take photos in back scattered electron (BSE) mode and secondary electron detect (SED) mode in the typical area;
- Obtain the main constituent elements and their proportion using energy dispersive spectroscopy (EDS) at the detection location.

**Figure 3:**
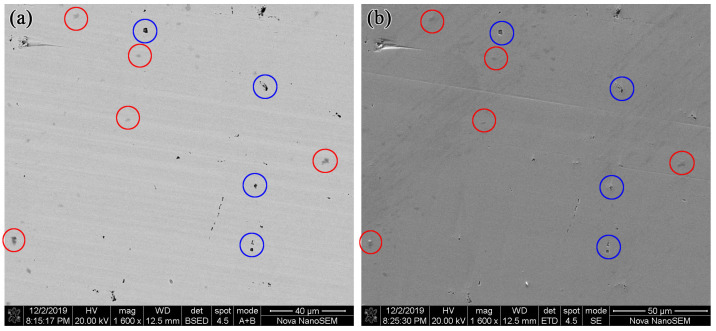
Pictures of SEM in the same detection scope with magnification of 1600 in Sample 1: (**a**) BSE mode. (**b**) SED mode.

In the measurement of SEM, the BSE mode was used to obtain the phase distribution and the SED mode was used to observe the surface topography. Through the comparison of the pictures taken in BSE mode and in SED mode, the phase composition of the test sample could be obtained. Figure 3 shows the pictures in BSE mode and SED mode of Sample 1 in the same detection scope with magnification of 1600.

**Figure 4:**
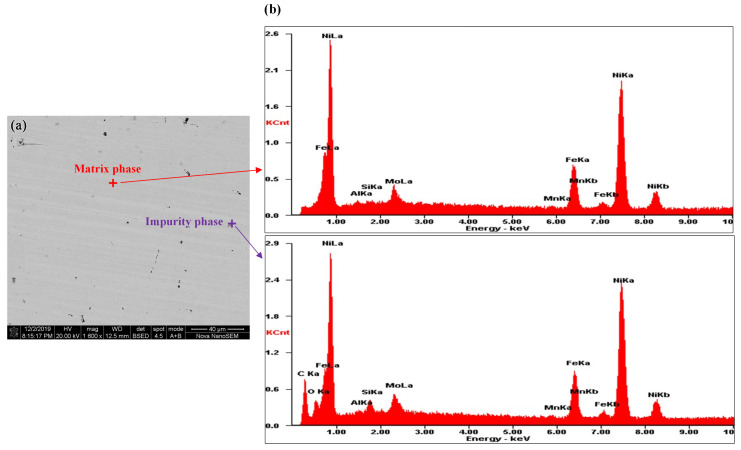
Phase distribution in Sample 1: (**a**) Pictures taken in BSE mode and the scanning points’ position. (**b**) Elemental spectrum.

From Figure 3a, it could be seen that there are three types of contrast in Sample 1, including light gray, dark gray (red circles), and black (blue circles). From Figure 3b, it could be seen that the black contrast represents the surface pits of the sample. So there are two phases in Sample 1, which are matrix phase and impurity phase. Then EDS is used to analyze the phases by scanning the chosen points, which is shown in Figure 4.

The element contents of the matrix phase and the impurity phase in Sample 1 are arranged in Table 4. It could be seen the main elements of the matrix phase in Sample 1 include Ni, Fe, and Mo, with the proportion of 80wt%, 15wt%, and 5wt%, respectively. Compared with the matrix phase, the impurity phase is clearly mixed with elements including C, O, and Si. It is inferred that the impurity phase should be a mixture of Ni/Fe carbide and ${\mathrm{SiO}}_{2}$, and ${\mathrm{SiO}}_{2}$ may originate from the abrasive in the polishing process. Since the relative content of impurity phase is quite low, the influence of the impurity phase could be ignored.

**Figure 5:**
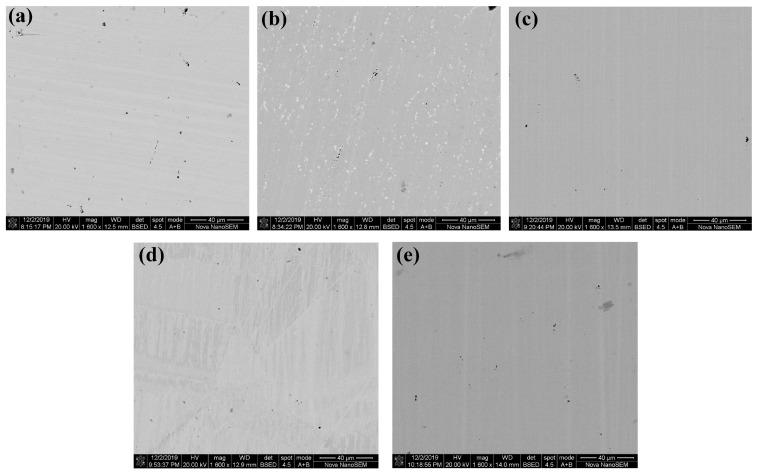
Pictures by SEM in BSE mode of all samples: (**a**) Sample 1. (**b**) Sample 2. (**c**) Sample 3. (**d**) Sample 4. (**e**) Sample 5.

The BSE pictures of all test samples are shown in Figure 5, and the analyzed compositions for the matrix phase of the samples are arranged in Table 5. It could be seen that the element type and content of each sample is essentially same except Sample 2, which contains a lot of Nb. Since Sample 2 and Sample 1 were provided by the same manufacturer, among which Sample 1 contains little Nb, the element of Nb may come from the annealing process. Despite this, the compositions of all samples are still similar, so the material composition is not the main reason on the difference in magnetic properties of the test samples.

#### 3.2.2. Texture Test

The testing procedure of the texture by the X-ray diffractomer (XRD) is listed as follows:

- Cut the test samples into appropriately sized pieces;
- Remove surface impurities of the sample pieces through polishing until the surface appears to be metallic luster;
- Fix the sample pieces using rubber putty as the sample holder and place the samples in XRD for detection;
- Analyze the XRD data and obtain the textures of the samples.

**Figure 6:**
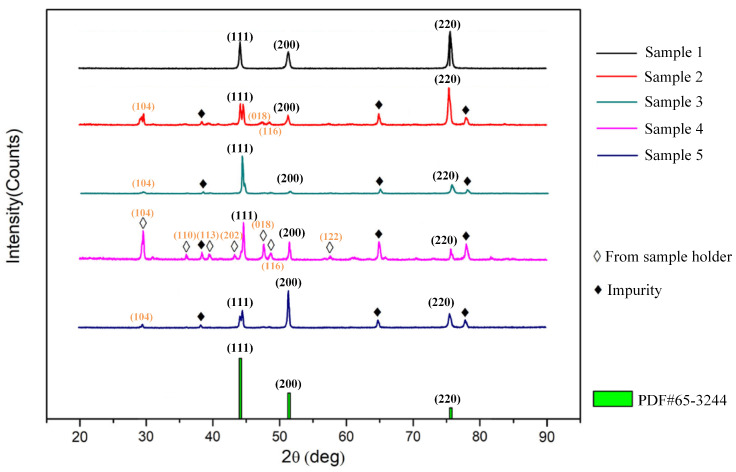
The test results of XRD.

The normalized test results of all samples are arranged in Figure 6.

It could be seen that all samples show high-intensity peaks in texture orientation (111), (200), and (220), which are consistent with the standard powder diffraction file (PDF) card of permalloy (No. 65-3244). Therefore, the samples by different manufacturers show similarity in texture. However, from Figure 6, it could be seen that there are some intensity peaks not containing in PDF card No. 65-3244 which are marked with diamond symbol. Comparing with the related PDF card, the intensity peaks marked with white diamond symbol came from the sample holder (calcium carbonate, PDF card No. 88-1807). The intensity peaks marked with black diamond symbol came from the impurities unable to find corresponding PDF card, which might be the sosoloid of Fe and Mo or Ni and Mo. Considering that the textures of all samples are similar, the texture contributes little on the difference in magnetic properties of the test samples.

#### 3.2.3. Grain Structure Test

The testing procedures of the grain structure by the confocal laser scanning microscope (CLSM) are listed as follows:

- Cut the test samples into appropriately sized pieces;
- Polish the surface of the sample pieces;
- Make chemical corrosion on samples’ surface to present grain morphology;
- Observe the grain morphology of each sample and calculate the size of grain.

Based on corrosion experience of steel materials and high-temperature alloy materials [30,31], three kinds of etchant were prepared for corrosion testing including 4% nitric acid alcohol solution, 4:10:20 copper sulfate/hydrochloric acid/water solution, and 1.8:10:20 iron chloride/hydrochloric acid/water solution.

A 4% nitric acid alcohol solution is usually used in the metallographic corrosion of steel materials. During the test, the permalloy was soaked in the 4% nitric acid alcohol solution for 5 min; there were no obvious signs of corrosion on the permalloy’s surface, which shows that the corrosion solution could not corrode the permalloy.

**Figure 7:**
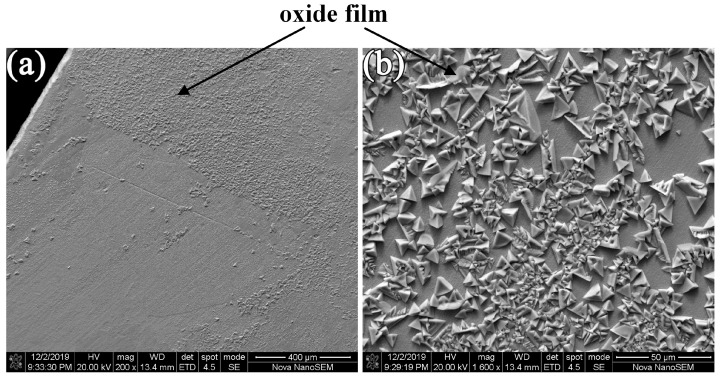
The SEM picture of permalloy surface corroded by 4:10:20 copper sulfate/hydrochloric acid/water solution: (**a**) magnification of 200. (**b**) magnification of 1600.

A 4:10:20 copper sulfate/hydrochloric acid/water solution is usually used in Ni-base superalloy. During the test, it was found that the 4:10:20 copper sulfate/hydrochloric acid/water solution was easy to form an oxide film on the surface of permalloy, which made it hard to distinguishing the grain structure, as shown in Figure 7.

**Figure 8:**
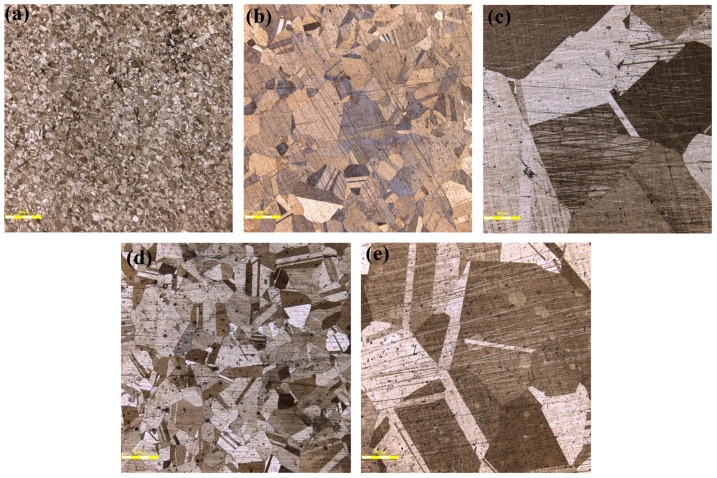
The grain structure of the test samples after corrosion by CLSM at magnification of 10: (**a**) Sample 1. (**b**) Sample 2. (**c**) Sample 3. (**d**) Sample 4. (**e**) Sample 5.

A 1.8:10:20 iron chloride/hydrochloric acid/water solution is another etchant for Ni-base alloy. During the test, the grain structure of permalloy was clearly presented after corrosion for about 1.5 min. So the 1.8:10:20 iron chloride/hydrochloric acid/water solution was chosen as the etchant for the test samples. The grain structure of the test samples after corrosion are observed by CLSM, which are shown in Figure 8.

It could be seen that the grain size of the test samples varies greatly, for the samples came from different manufacturers with different annealing processes. The grain size of each sample was measured by the intersection point method according to the measurement standard GB/T 6394-2017 [32].

The procedures are listed as follows:

- Prepare the representative picture taken by CLSM for grain size calculation;
- Draw three circles with Radius $R_{1}$, $R_{2}$, and $R_{3}$ on the picture (the biggest circle should be as large as possible within the scope of the picture);
- Calculate the intersection points’ number of the grains and the circles $N_{i}$;
- Obtain the average grain size $\bar{L}$ according to the equation below:
  (17) $$\bar{L}=\frac{L}{M\cdot N_{i}}$$
  where *L* is the sum of circumference of the three circles and *M* is the magnification.

**Figure 9:**
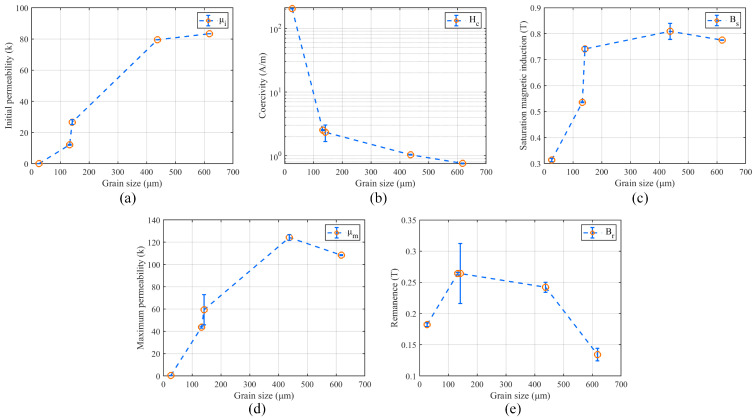
The test results of the grain size and the magnetic properties: (**a**) Grain size and initial permeability. (**b**) Grain size and coercivity. (**c**) Grain size and saturation magnetic induction. (**d**) Grain size and maximum permeability. (**e**) Grain size and remanence.

The grain size of each sample is arranged in Table 6 and the relation between the grain size and the magnetic properties from the test results is shown in Figure 9. The test result shows that the grain size is closely related to the initial permeability and the coercivity, which is consistent with the theory.

## 4. Discussion

**Figure 10:**
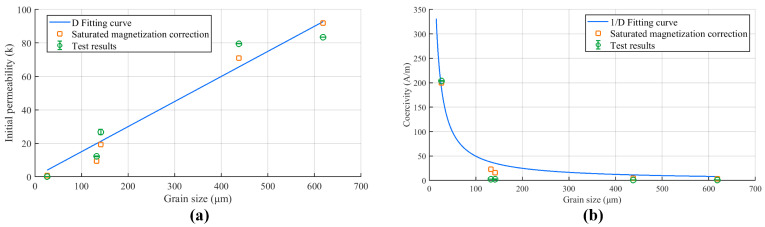
The comparison the test results, the fitting curve and the saturated magnetization correction for the fitting curve: (**a**) Grain size and initial permeability. (**b**) Grain size and coercivity.

The test method of permalloy including the material composition, the texture, and the grain structure is proposed are described in the previous section of this paper. According to the test result of different samples, the grain structures are closely related to the magnetic properties. The theory shows that the initial permeability is proportional to the grain size ($\mu_{i}\;\;∼\;\;D$) and the coercivity is inversely proportional to the grain size ($H_{c}\;\;∼\;\;1/D$) according to Equations (10) and (16). To explore the relation between the theory and the experiments, the D fitting curve and 1/D fitting curve are introduced for comparison, which are shown in Figure 10. Considering the saturation magnetic induction for each sample is different, which is proportional to the saturated magnetization ($B_{s}\;\;∼\;\;M_{s}$), the saturated magnetization correction for the fitting curve is also drawn in Figure 10.

It could be seen that the fitting curve could clearly describe the variation trend of the grain size with the initial permeability and the coercivity from test results, which proves the consistency between theory and experiment. Meanwhile, after saturated magnetization correction, the fitting points are closer to the test data, which verifies the correctness of Equations (10) and (16). Other magnetic properties such as the saturation magnetic induction, the maximum permeability and the remanence are related to the inherent attributes of the material, which are comprehensive and hard to observe. So the change patterns of the other magnetic properties are difficult to obtain, which are not discussed in the manuscript. Since the initial permeability and the coercivity could directly reflect the performance of the permalloy, which could be observed directly from the grain size, the method of evaluating the property of the high-permeability material has been broadened. Meanwhile, the procedure of polishing and chemical corrosion could be easily completed by automated devices, which brings the promotion of the efficiency comparing with the soft magnetic testing instruments.

## 5. Conclusions

This paper proposes a novel test method of high-permeability material’s microstructure including the material composition, the texture and the grain structure to reflect the high-permeability material’s magnetic properties. The relationship between the microstructure and the magnetic properties of the high-permeability material is derived and calculated through the magnetic domain theory using MFE to explain the pinning effect for the first time. Through the experiment, the relationship between the microstructure and the magnetic properties is proved to be clear and credible, which provides a new approach of evaluating the property of the high-permeability material. Compared with typical magnetic testing methods, the proposed method of microstructure to obtain the magnetic properties will promote the detection efficiency and reduce the detection complexity in some detection cases. It has important significance in the high efficiency sampling inspection for industrial applications.

## Figures and Tables

**Table 1:** The information of the test samples.

Sample No.	Sample Brand	Sample State	Manufacturer
Sample 1	1J85	unannealed	Shanghai (DaiXin)
Sample 2	1J85	annealed *	Shanghai (DaiXin)
Sample 3	1J85	annealed *	Beijing (BeiYe)
Sample 4	1J85	annealed *	Beijing (BeiYe)
Sample 5	Mumetal	annealed *	Germany (VAC)

* Different annealing processes are tried by different manufacturers.

**Table 2:** The test parameters of the test samples for magnetic properties measurement.

Sample No.	Thickness—One Piece (mm)	Thickness—Laminations C (mm)	Outer Diameter A (mm)	Inner Diameter B (mm)	Weight (g)
Sample 1	2.5	5.07	39.98	31.90	19.6
Sample 2	1	2.83	40.00	32.05	10.4
Sample 3	1	2.90	39.95	31.57	11.8
Sample 4	2	6.24	40.04	31.98	23.8
Sample 5	0.75	6.11	28.50	19.96	16.7

**Table 3:** The test results of magnetic properties.

Sample No.	Initial Permeability $\mu_{i}$ (k)	Maximum Permeability $\mu_{m}$ (k)	Saturation Magnetic Induction $B_{s}$ (T)	Remanence $B_{r}$ (T)	Coercivity $H_{c}$ (A/m)
Sample 1	0.134 ± 0.011	0.430 ± 0.008	0.314 ± 0.006	0.182 ± 0.003	203.550 ± 0.531
Sample 2	12.130 ± 0.269	43.745 ± 0.829	0.536 ± 0.001	0.264 ±0.002	2.516 ±0.047
Sample 3	83.395 ± 0.045	108.350 ± 0.367	0.775 ± 0.001	0.134 ± 0.010	0.757 ± 0.008
Sample 4	26.675 ± 1.571	59.395 ± 13.615	0.741 ± 0.009	0.264 ± 0.048	2.347 ± 0.683
Sample 5	79.505 ± 0.037	124.250 ± 2.572	0.809 ± 0.031	0.242 ± 0.008	1.038 ± 0.013

**Table 4:** The element contents of the matrix phase and the impurity phase in Sample 1.

Phase	Ni (wt%)	Fe (wt%)	Mo (wt%)	Mn (wt%)	Al (wt%)	Si (wt%)	C (wt%)	O (wt%)
Matrix phase	78.21	14.73	5.70	0.50	0.58	0.27	0	0
Impurity phase	53.56	10.43	3.54	0.50	0.19	1.16	26.81	3.81

**Table 5:** The element contents of the matrix phase in each test sample.

Samp No.	Ni (wt%)	Fe (wt%)	Mo (wt%)	Mn (wt%)	Al (wt%)	Si (wt%)	Nb (wt%)
Sample 1	78.21	14.73	5.70	0.50	0.58	0.27	0
Sample 2	78.14	11.02	2.82	0.69	0.69	0.52	6.12
Sample 3	78.89	14.58	5.60	0.46	0.23	0.24	0
Sample 4	78.39	14.34	5.67	0.53	0.61	0.46	0
Sample 5	78.34	15.91	4.16	0.64	0.46	0.48	0

**Table 6:** The grain size of the test samples.

Sample No.	Grain Size (μm)
Sample 1	26.2
Sample 2	132.4
Sample 3	618.4
Sample 4	141.6
Sample 5	437.7

## Data Availability

Not applicable.
